# Family caregivers' experience with healthcare and social care professionals and their participation in health checkups: A cross sectional study in Japan

**DOI:** 10.1002/jgf2.599

**Published:** 2022-12-14

**Authors:** Gen Nakayama, Shoichi Masumoto, Junji Haruta, Tetsuhiro Maeno

**Affiliations:** ^1^ Department of Primary Care and Medical Education, Faculty of Medicine University of Tsukuba Tsukuba Japan; ^2^ Department of Family Medicine, General Practice and Community Health, Faculty of Medicine University of Tsukuba Tsukuba Japan; ^3^ Department of General Medicine Tsukuba Central Hospital Ushiku Japan; ^4^ Medical Education Center, School of Medicine Keio University Tokyo Japan

**Keywords:** caregivers, health behavior, healthcare quality assurance, health services evaluation, person‐centered care, primary health care

## Abstract

**Background:**

For family caregivers, who are generally regarded as a vulnerable population, having regular checkups is a desirable health behavior. This study examined family caregivers' habit of having regular checkups prior to becoming involved with professionals who care for patients, and whether they had had recent checkups. We then examined the association between family caregivers' experience with professionals and their participation in checkups after adjusting for the past habit.

**Methods:**

We conducted a cross sectional survey in Japan between November and December 2020. We recruited family caregivers who were aged 40–74 years and caring for community‐dwelling adult patients. The outcome variable was whether family caregivers had undergone any health checkups since April 2019. We assessed family caregivers' experience using the Japanese version of the Caregivers' Experience Instrument (J‐IEXPAC CAREGIVERS).

**Results:**

Of the 1091 recruited family caregivers, 629 were included in the analysis. Of these, 358 had previously undergone regular checkups, and 158 had no checkups or selected the option “unknown.” Outcome rates in each group were 74.6% and 43.0%, respectively, and 62.0% for all 629 caregivers. Multivariate modified Poisson regression analysis revealed that among the J‐IEXPAC CAREGIVERS scores, only the domain score for *attention for the caregiver* was significantly associated with family caregivers' participation in checkups (adjusted prevalence ratio per 1 SD increase = 1.07; 95% CI 1.01–1.14).

**Conclusions:**

Among family caregivers' experience with professionals, the factor that focused on caregivers themselves was significantly associated with their participation in checkups. This finding underscores the significance of caregiver‐focused care.

## Graphical abstract

This study examined the association between family caregivers' experience with professionals and their participation in health checkups after adjusting for multiple possible confounders, including a past habit of having regular checkups. Among family caregivers' experience with professionals, the factor that focused on caregivers themselves was significantly associated with their participation in checkups. Our findings should encourage healthcare and social care professionals to try to improve family caregivers' experience by focusing on the caregivers themselves.

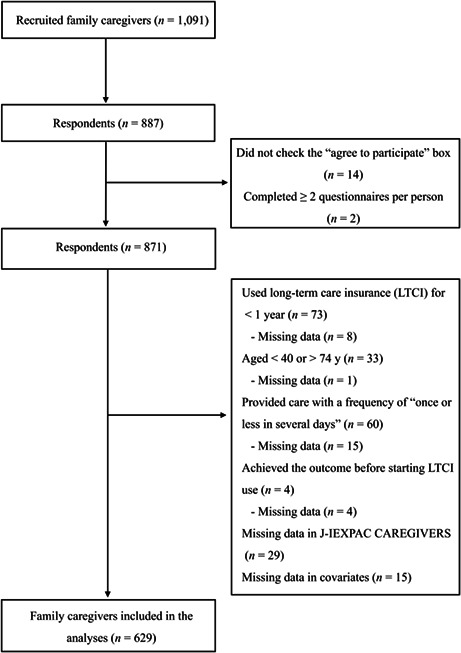

## 1 INTRODUCTION

The rapidly aging world population and increasing incidence of chronic diseases is leading to a rise in the roles and needs of family caregivers as elderly patients (care recipients) increasingly rely on family members to support their daily activities.^1^ Previous studies have reported that family caregivers have more problems with their psychological or physical health than noncaregivers.^2^, ^3^ Family caregivers are also reported to underutilize needed healthcare services,^4^ and to tend to subordinate their own health needs to those of others.^5^ Therefore, family caregivers are a population that warrants a strategic public health approach. Indeed, the Centers for Disease Control and Prevention (CDC) has released several calls to action in support of family caregivers.^6^ These include encouraging family caregivers to have regular health checkups.^6^

Having regular health checkups is a desirable health behavior for family caregivers.^7^, ^8^ Although the effectiveness of checkups in the general population is controversial,^9^, ^10^, ^11^ checkups have been associated with controlling risk factors, incorporating preventive services, and improving patient‐reported outcomes,^12^ and are therefore considered particularly appropriate for vulnerable populations, such as those with low self‐rated health and poor connection to primary care.^12^ Family caregivers represent such a vulnerable group,^2^, ^3^, ^4^, ^5^ and having regular checkups is therefore a desirable health behavior. In countries like Japan and South Korea, where the government already recommends regular checkups for the general population, special attention is considered necessary to encourage family caregivers to undergo health checkups.^13^, ^14^

A recent examination of health checkups among family caregivers found that family caregivers who had more positive experiences with healthcare and social care professionals – in other words, positive interactions between the caregiver and the professionals who care for patients – were more likely to participate in health checkups.^15^ This concept of experience has attracted attention as a means of evaluating the quality of professional care from the caregiver's perspective.^16^, ^17^ The authors suggested^15^ that family caregivers with more positive experiences received more emotional support for their own health and well‐being from professionals, and as a result may have paid more attention to their own health and tended to participate in checkups; to our knowledge, the study is the first to suggest an association between family caregiver experience with professionals who care for patients and their participation in health checkups. Nevertheless, it has some limitations. First, the study was a secondary analysis of data from scale development, and the sample size was small. Second, there were potential unmeasured confounders. In particular, the study did not consider family caregivers' habit of having regular checkups prior to becoming involved with professionals who care for patients. Because such past habits may have a significant impact on participation in current checkups, we believe that adjusting for a past habit may lead to a more robust examination.

In this study, we first examined family caregivers' habit of having regular checkups prior to becoming involved with professionals who care for patients with chronic conditions, and whether they had had recent checkups. We then examined the association between family caregivers' experience with these professionals and their participation in health checkups after adjusting for possible confounders, including a past habit of having regular checkups.

## 2 METHODS

### 2.1 Design, setting, participants and procedures

We conducted a cross sectional survey, the Caregivers' own Health And their eXperience of care Ibaraki Survey (CaregiversHAXIS), in Ibaraki Prefecture, located northeast of Tokyo, in November and December, 2020. We recruited family caregivers who were caring for community‐dwelling adult patients under the supervision of care managers working in three municipalities in Ibaraki (Appendix S1). Under Japan's Long‐Term Care Insurance (LTCI) system, care managers support adult patients and their family caregivers by managing and coordinating the roles of other healthcare and social care professionals.^18^ The researchers instructed the care managers to recruit family caregivers consecutively, in order of their original appointments with patients and family caregivers. To be eligible, family caregivers were required to be caring for patients who were suffering from “chronic conditions.”^19^

All data on family caregivers were collected using a self‐administered questionnaire. The family caregivers provided informed consent via the questionnaires and directly returned the questionnaires by mail to the office of our university.

#### 2.1.1 Inclusion criteria

Study participants were eligible for this study if they were aged 40–74 years and had been using LTCI for ≥1 year. The Japanese government places particular emphasis on health checkups for people aged 40–74 years.^20^

#### 2.1.2 Exclusion criteria

Family caregivers were excluded from the study if they answered ≥2 questionnaires per person, because caregivers caring for two or more people were instructed to limit their responses to the care of the most dependent patient; provided care with a frequency of “once or less in several days,”^21^ because family caregivers who provide less frequent care are reported to have limited contact with professionals^17^; or had undergone any health checkups since April 2019 but before starting LTCI use, because the time at which family caregivers began engaging with professionals was chosen to represent the time of the first LTCI use.

### 2.2 Measures

#### 2.2.1 Outcome variable: Participation in health checkups

The outcome variable was whether family caregivers had undergone a health checkup since April 2019. Based on the Comprehensive Survey of Living Conditions (CSLC) questionnaire administered by the government,^22^ we used the following question to determine if caregivers had undergone a health checkup: “Have you had any health checkups (a health checkup or a thorough medical checkup) since April 2019?” This question excluded dental checkups, examinations to diagnose a suspected disease or follow‐up, or screening for cancer only.^22^ While the CSLC questionnaire typically asks about participation in health checkups “in the past year,” we modified the time period to “since April 2019” to include family caregivers who may not have undergone a health checkup “in the past year” owing to the effects of coronavirus disease 2019. In Japan, the annual health checkup system covers the period from April to March.

#### 2.2.2 Explanatory variable: Family caregivers' experience with healthcare and social care professionals who care for patients

The Japanese version of the Caregivers' Experience Instrument (J‐IEXPAC CAREGIVERS)^23^ was used to assess family caregivers' experience. IEXPAC CAREGIVERS focuses on the interaction between family caregivers and professionals who care for patients with chronic conditions.^16^ This scale was developed and validated in Spain, based on the Instrument to evaluate the EXperience of PAtients with Chronic diseases (IEXPAC), which was in turn theoretically based on the Chronic Care Model (CCM).^24^ Because the CCM places importance on the systematization of primary care,^25^ IEXPAC scales were designed to evaluate a range of professionals, rather than any specific one.

J‐IEXPAC CAREGIVERS evaluates professionals such as primary care physicians, nurses and care managers from the family caregiver's perspective. This scale consists of two dimensions—*attention for the patient* and *attention for the caregiver*. The former dimension captures the process by which professionals work with caregivers to provide care for patients and the latter captures the process by which professionals provide care to family caregivers as co‐clients. Each item is rated on a five‐point Likert scale ranging from 1 (Never) to 5 (Always). A scale score is calculated by simply summing the scores for each of 12 items, with total scores ranging from 12 to 60. A higher total score indicates higher quality of integrated care from the caregiver's perspective. The internal consistency (Cronbach's alpha) determined in the J‐IEXPAC CAREGIVERS development study was 0.92.^23^

#### 2.2.3 Covariates

Based on studies that examined factors that affect participation in disease screening or health promotion behaviors among family caregivers,^14^, ^26^, ^27^ we included the following as possible confounders in the association between family caregivers' experience and their participation in health checkups: age, gender, relationship with care recipient, self‐rated health, type of insurance, educational attainment, annual household income, municipality of residence, caregiving time, caregivers’ experience as patients (caregivers' PX), social support by relatives or acquaintances and participation behavior in health checkups before starting LTCI use. Caregiving time was determined using a question based on the CSLC questionnaire.^21^ Because daily caregiving time strongly reflects patient deficiencies in activities of daily living,^28^ we did not include the patients' functional status as a covariate in the present study. Caregivers' PX was measured using the Japanese version of Primary Care Assessment Tool Short Form (JPCAT‐SF).^29^ We first assessed whether the subject had a usual source of care and, if so, measured PX in primary care with the overall JPCAT‐SF score. To determine social support by relatives or acquaintances, we measured emotional support, which has in particular been shown to be associated with preventive health behaviors.^30^, ^31^ Because the breadth of our questionnaire was limited, we used the following item to determine emotional support based on previous research^32^: “I have relatives or acquaintances who listen to my worries and fears (other than the professionals who care for me and my loved ones).” Participants chose from responses ranging from 1 (Disagree) to 5 (Agree).

### 2.3 Statistical analysis

We reported the characteristics of family caregivers and the distribution of the J‐IEXPAC CAREGIVERS score. We also described information about participation in health checkups before and whether they had had recent checkups. A robust (modified) Poisson regression model was used to determine whether the J‐IEXPAC CAREGIVERS total score and each domain score were positively associated with participation in health checkups. We analyzed the unadjusted association between J‐IEXPAC CAREGIVERS scores and outcome by calculating the crude prevalence ratio in bivariate regression.

In the multivariate regression, the variables described in “Covariates” were included as possible confounders. All variables except J‐IEXPAC CAREGIVERS score and age were divided into multiple categorical variables. Municipality of residence was categorized into two groups (Appendix S1). Caregivers' PX was categorized into four groups: no usual source of care or unknown, and tertiles of JPCAT‐SF total score. Social support by relatives or acquaintances was categorized into three groups: disagree or partly disagree, neither agree nor disagree, and partly agree or agree, based on a previous study.^31^ Participation behavior in health checkups before starting LTCI use was categorized into the following three groups by combining the “no participation” and “unknown” options into one category: regular participation, occasional participation, and no participation or unknown. We included each J‐IEXPAC CAREGIVERS score separately in the model and interpreted the results without Bonferroni correction. Participants with missing data were excluded from all analyses. Since the use of logistic regression analysis was planned during the study planning stage, the sample size setting and the results of logistic regression analysis are presented as a supplement (Appendix S2). Statistical analyses were conducted using SPSS Statistics version 28 (IBM Corp.).

## 3 RESULTS

### 3.1 Participants' characteristics and descriptive analysis of the J‐IEXPAC CAREGIVERS score

**FIGURE 1:**
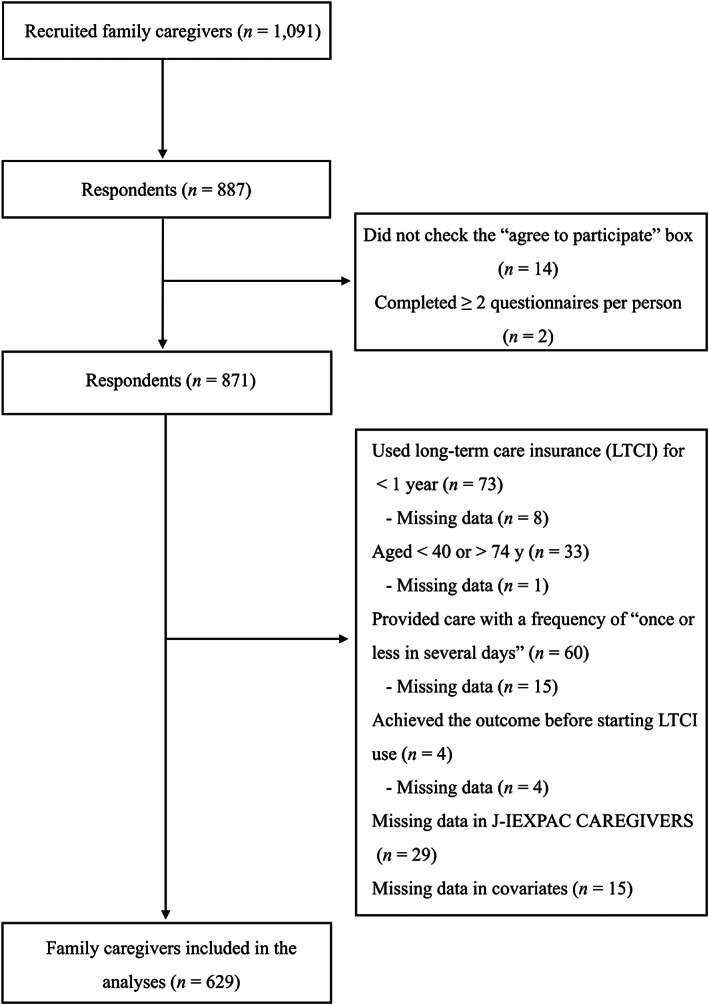
Flow chart of participants. J‐IEXPAC CAREGIVERS, Japanese version of the Caregivers' Experience Instrument.

Of the 1091 recruited family caregivers, 887 (81.3%) responded to the questionnaire. Figure 1 shows a flow chart of the study participants, with 629 (57.7% of 1091) ultimately included in the analysis. Table 1 shows the characteristics of the 629 family caregivers. Median age was 62 years, and the majority were women (74.7%).

**TABLE 1:** Family caregivers' characteristics (*N* = 629)

Characteristic	Total
	(*N* = 629)
Gender, *N* (%)	
Men	159 (25.3)
Women	470 (74.7)
Age (years), median [IQR]	62 [56–68]
Relationship with care recipient	
Spouse	120
Child	375
Child‐in‐law	111
Parent	7
Sibling	6
Other	10
Self‐rated health, *N* (%)	
Poor	34 (5.4)
Not very good	112 (17.8)
Good	368 (58.5)
Very good	115 (18.3)
Insurance type, *N* (%)	
National insurance	329 (52.3)
Employee's insurance, employees	158 (25.1)
Employee's insurance, dependents	126 (20.0)
Other	16 (2.5)
Education, *N* (%)	
Did not complete high school	20 (3.2)
High school	284 (45.2)
Career college, junior college, or higher professional school	191 (30.4)
College or graduate school	134 (21.3)
Annual household income (million JPY), *N* (%)	
<2.50 (about USD 24,000)	219 (34.8)
2.50–4.99	246 (39.1)
5.00–7.99	103 (16.4)
≥8.00	61 (9.7)
Municipality of residence, *N* (%)	
One of the three municipalities where recruitment was conducted	541 (86.0)
Other	88 (14.0)
Social support by relatives or acquaintances^a^, *N* (%)	
Disagree or partly disagree	103 (16.4)
Neither agree nor disagree	69 (11.0)
Partly agree or agree	457 (72.7)
Caregiving time per day, *N* (%)	
Almost all day	114 (18.1)
Half day	113 (18.0)
2–3 h	110 (17.5)
Lend a hand when needed	292 (46.4)
Caregivers' PX, *N* (%)	
No usual source of care or unknown	244 (38.8)
Lower tertile (Tertile 1)^b^	134 (21.3)
Middle tertile (Tertile 2)^b^	125 (19.9)
Higher tertile (Tertile 3)^b^	126 (20.0)

Abbreviations: IQR, interquartile range; PX, patient experience; LTCI, long‐term care insurance.

^a^ “I have relatives or acquaintances who listen to my worries and fears (other than the professionals who care for me and my loved ones).”

^b^ Tertile 1, 2.1 to 33.3; Tertile 2, 33.4 to 47.9; Tertile 3, 48.0 to 100.

Table 2 shows the mean and standard deviation (SD) of the J‐IEXPAC CAREGIVERS total score and the proportion of family caregivers who responded “always” or “almost always” to each J‐IEXPAC CAREGIVERS item. The average J‐IEXPAC CAREGIVERS total score was 40.7 (SD 8.3) out of 60 points.

**TABLE 2:** Japanese version of the Caregivers' experience instrument (J‐IEXPAC CAREGIVERS) score and the proportion of family caregivers who selected “always” or “almost always” to each J‐IEXPAC CAREGIVERS item (*N* = 629).

	Mean (SD)
Total score	40.7 (8.3)
Domain scores	
Attention for the patient (items 1, 2, 4, 5, 6, 7, and 8)	26.1 (5.0)
Attention for the caregiver (items 9, 10, 11, and 12)	12.5 (3.3)
	**Always/almost always responses (%)**
1. They respect the lifestyle of the person I care for	72.7
2. They are coordinated to offer us good care	52.9
3. They help me become informed via the Internet	12.2
4. I now know how to look after them better	43.1
5. They ask me about and help me follow the treatment plan of the person in my care	73.0
6. We agree on the most important objectives of their care to control their health problems better	67.9
7. They ensure that he/she takes the medication correctly	47.5
8. They are concerned about the wellbeing of the person in my care	74.1
9. They are concerned about my health and well being	56.4
10. They are concerned about my emotional and physical burden	49.0
11. They inform me about health and social resources that can help me	49.8
12. They encourage me to talk to other caregivers	6.0

*Note*: J‐IEXPAC CAREGIVERS evaluates a range of professionals such as primary care physicians, nurses and care managers, rather than any specific one. The items listed in this table are written as they are in the original English version of IEXPAC CAREGIVERS. The text of each item shows only a portion of the original, and the full text is available online (http://www.iemac.es/iexpac/index.php). IEXPAC©2015.

Abbreviation: SD, standard deviation.

### 3.2 Family caregivers' past habit of having regular checkups and participation in recent checkups

Table 3 shows the distribution of family caregivers by past habit of having regular checkups, and participation in recent checkups. Sixty‐two percent of all 629 caregivers indicated that they had participated in a health checkup since April 2019. Of the 358 family caregivers who had been in the habit of having regular checkups, 91 (25.4%) caregivers had not undertaken a recent checkup. On the other hand, of the 158 family caregivers who had no checkups or selected the option “unknown,” 68 (43.0%) had participated in a recent checkup.

**TABLE 3:** Family caregivers' past habit of having regular checkups and participation in recent checkups (*N* = 629).

	Participation in recent checkups^c^		
	No	Yes	Total
Past habit^a^			
Regular participation	91 (25.4%)	267 (74.6%)	358
Occasional participation	58 (51.3%)	55 (48.7%)	113
No participation or unknown^b^	90 (57.0%)	68 (43.0%)	158
Total	239 (38.0%)	390 (62.0%)	629

^a^ Participation behavior in health checkups before starting long‐term care insurance use.

^b^ Those who had no checkups (*n* = 140) or selected the option “unknown” (*n* = 18).

^c^ Participation in health checkups since April 2019.

### 3.3 Associations of J‐IEXPAC CAREGIVERS score with family caregiver participation in health checkups

Table 4 shows the results of bivariate and multivariate modified Poisson regression analyses of the association of J‐IEXPAC CAREGIVERS scores with family caregivers' participation in health checkups. In bivariate (unadjusted) models, the J‐IEXPAC CAREGIVERS total score and the domain score for *attention for the caregiver* were significantly associated with family caregivers' participation in health checkups. After adjusting for possible confounders, only the domain score for *attention for the caregiver* was positively associated with family caregivers' participation in health checkups (adjusted prevalence ratio per 1 SD increase = 1.07; 95% CI 1.01–1.14).

**TABLE 4:** Associations of the Japanese version of the Caregivers' Experience Instrument (J‐IEXPAC CAREGIVERS) scores with family caregiver participation in health checkups (*N* = 629).

	Bivariate model^a^	*p* Value	Multivariate model^a^ ^,^ ^b^	*p* Value
	Crude PR (95% CI)^c^		Adjusted PR (95% CI)^c^	
J‐IEXPAC CAREGIVERS				
Total score	1.07 (1.01–1.14)	0.026	1.06 (0.99–1.12)	0.079
Domain scores				
Attention for the patient	1.06 (1.00–1.13)	0.051	1.04 (0.98–1.11)	0.205
Attention for the caregiver	1.08 (1.01–1.15)	0.012	1.07 (1.01–1.14)	0.028

Abbreviations: CI, confidence interval; PR, prevalence ratio.

^a^ Each score was included separately in the model.

^b^ All three models were adjusted for age, gender, relationship with care recipient, self‐rated health, insurance type, education, annual household income, municipality of residence, social support by relatives or acquaintances, caregiving time per day, caregivers' experience as patients (caregivers' PX), and participation behavior in health checkups before initiation of long‐term care insurance use.

^c^ Per 1 SD (standard deviation) increase.

## 4 DISCUSSION

We first examined family caregivers' habit of having regular checkups prior to becoming involved with professionals who care for patients, and whether they had had recent checkups. We found that 25.4% of family caregivers who had been in the habit did not undertake a recent checkup, whereas 43% of family caregivers who had no checkup habit (including those who selected “unknown”) had received a recent checkup. We then examined the association between family caregivers' experience with professionals, as measured by the J‐IEXPAC CAREGIVERS score, and their participation in recent checkups after adjusting for a past habit of having regular checkups. Results showed a significant association only for the domain score *attention for the caregiver* among the J‐IEXPAC CAREGIVERS scores. Our findings reinforce the importance of enhancing care that focuses on family caregivers within the context of their having checkups as their own care.

The results of past habits and recent health checkups in family caregivers can be explained by the transtheoretical model of behavior change (TTM), which describes the following stages of change: precontemplation, contemplation, preparation, action, maintenance and termination.^33^ We found that 25.4% of family caregivers who had been in the habit of having regular checkups did not have recent checkups. This finding is similar to that of previous research conducted in the U.S., which showed that while middle‐aged and older family caregivers engaged in 86% of recommended preventive health practices, including blood pressure and cholesterol screenings, in the past, they currently conduct only 63% of these practices.^34^ According to TTM, even for people who have reached the stage of maintaining their desired behavior, the temptation to step back is very strong.^35^ Triggers for this temptation include negative affect or emotional distress, and social situations. Our present and previous findings^34^ suggest that caregiving responsibilities may also be a trigger. On the other hand, we found that 43% of family caregivers who reported not having a checkup habit (including those who selected “unknown”) had engaged in recent checkups. These family caregivers may have previously been at a stage prior to “action,” but their caregiving responsibilities may have led them to become concerned about their health and consequently to take action (i.e., having checkups). Family caregivers who participated in a qualitative survey perceived their self‐care to be negatively affected by caregiving and, in response to a question about the things they considered to be important in self‐care, reported that having regular checkups was one way for them to maintain their health.^36^ Thus, caregiving responsibilities may have conflicting effects on the family caregivers' decision to have checkups.

After controlling for variables related to caregiving responsibilities and a previous habit of having regular checkups, the only score significantly associated with participation in health checkups was *attention for the caregiver*. This differs from the results of a previous preliminary analysis, in which all scores were significantly associated with participation in health checkups.^15^ The significance of the domain score for *attention for the caregiver* was common in both studies, which is theoretically understandable because the main construct of this domain is the family caregiver's perception of whether the caregiver has professional care support for his or her own health and well‐being, and to obtain information to help acquire such support. Interpersonal relationships in facilitating and preserving well‐being prevents and buffers stress; increases connectedness, control and self‐esteem; and consequently promotes health behaviors.^37^ On the other hand, the domain score for *attention for the patient* was significant in the previous study but showed no significance in the present study. In the previous study, it was noted that this domain score may be significantly associated with participation in health checkups because the two domain scores for *attention for the patient* and *attention for the caregiver* showed high correlation. In the present study, there was a gap in caregivers' recognition of these two compared to the previous study (Spearman's rank correlation coefficient for both domains: *r* = 0.698 in the present study, *r* = 0.791 in the previous study), which may have led to the lack of a significant association in the domain score for *attention for the patient*. Furthermore, the J‐IEXPAC CAREGIVERS total score did not show significance in this study, probably because the domain score for *attention for the patient* accounts for a larger proportion of the total score (Table 2). Given that the previous study was conducted using a convenience sample of limited size, versus the relatively large consecutive sample in the present study, our present results may be more reflective of the population.

The strength of the current study is that, compared with the previous study,^15^ its more robust comparison shows that the domain score for *attention for the caregiver* is associated with participation in checkups. This result in turn suggests that caregiver‐focused care may have a positive impact on having checkups, even though caregiving responsibilities may have conflicting effects on family caregivers' decision to have checkups. Our findings should encourage healthcare and social care professionals to try to improve family caregivers' experience by referring to the items that comprise the *attention for the caregiver* domain of J‐IEXPAC CAREGIVERS, in the context of caregivers being more aware of care for their own health.

Our study also has several potential limitations. First, the results of past habits and recent health checkups in family caregivers were obtained from descriptive statistics, and no comparison with the control group was made. Second, we did not measure social support by relatives or acquaintances using validated measures of psychometric properties. Third, we did not include basic information on the patient (care recipient) in this article, out of respect for the ethical challenge we faced when attempting to obtain direct patient consent at collection. However, as noted in the Methods section, we believe that we were able to adjust confounders appropriately using caregiving time, which reflects patient deficit in activities of daily living. Fourth, the study design was cross sectional, and thus a causal relationship could not be definitively established. However, because we collected time‐sensitive data on the timing of family caregivers' engagement with professionals and the occurrence of outcomes after this engagement, we believe that reverse causality is unlikely.

## 5 CONCLUSION

We found that one‐quarter of family caregivers who had been in the habit of having regular checkups had not undertaken a recent checkup, whereas approximately 40% of family caregivers who did not have a checkup habit had received a recent checkup. Further analysis revealed that, among family caregivers' experience with professionals, the factor that focused on family caregivers themselves was significantly associated with their participation in checkups. Our findings reinforce the significance of family caregivers' experience, especially in terms of the receipt of care by caregivers themselves, in the context of having checkups as a component of their own care.

## ETHICS APPROVAL

The study was approved by the Ethics Committee of the Faculty of Medicine, University of Tsukuba (approval no. 1518‐2). This committee grants approval to studies based on the provisions of the Declaration of Helsinki and Japanese ethical guidelines (Ethical Guidelines for Medical and Health Research Involving Human Subjects).

## PATIENT CONSENT STATEMENT

All participants were volunteers and checked the box on the questionnaire indicating their intention to participate.

## CLINICAL TRIAL REGISTRATION

None.

## CONFLICT OF INTEREST

The authors have stated explicitly that there are no conflicts of interest in connection with this article.

## Supporting information


Appendix S1‐S2

## References

[1] Jika BM , Khan HTA , Lawal M . Exploring experiences of family caregivers for older adults with chronic illness: a scoping review. Geriatr Nurs. 2021;42(6):1525–32. 10.1016/j.gerinurse.2021.10.010 34735999

[2] Roth DL , Perkins M , Wadley VG , Temple EM , Haley WE . Family caregiving and emotional strain: associations with quality of life in a large national sample of middle‐aged and older adults. Qual Life Res. 2009;18(6):679–88. 10.1007/s11136-009-9482-2 19421895 PMC2855243

[3] Pinquart M , Sörensen S . Differences between caregivers and noncaregivers in psychological health and physical health: a meta‐analysis. Psychol Aging. 2003;18(2):250–67. 10.1037/0882-7974.18.2.250 12825775

[4] Tingey JL , Lum J , Morean W , Franklin R , Bentley JA . Healthcare coverage and utilization among caregivers in the United States: findings from the 2015 behavioral risk factor surveillance system. Rehabil Psychol. 2020;65(1):63–71. 10.1037/rep0000307 31971434

[5] Collins LG , Swartz K . Caregiver care. Am Fam Physician. 2011;83(11):1309–17. 21661713

[6] Centers for Disease Control [CDC] . Caregiving for family and friends: a public health issue Washington, DC. 2018 [cited 2022 May 26]. Available from: https://www.cdc.gov/aging/agingdata/docs/caregiver‐brief‐508.pdf

[7] Peters M , Rand S , Fitzpatrick R . Enhancing primary care support for informal carers: a scoping study with professional stakeholders. Health Soc Care Community. 2020;28(2):642–50. 10.1111/hsc.12898 31770820 PMC7027470

[8] Oliveira D , Sousa L , Orrell M . Improving health‐promoting self‐care in family carers of people with dementia: a review of interventions. Clin Interv Aging. 2019;14:515–23. 10.2147/CIA.S190610 30880932 PMC6402440

[9] Si S , Moss JR , Sullivan TR , Newton SS , Stocks NP . Effectiveness of general practice‐based health checks: a systematic review and meta‐analysis. Br J Gen Pract. 2014;64(618):e47–53. 10.3399/bjgp14X676456 24567582 PMC3876170

[10] Krogsbøll LT , Jørgensen KJ , Gøtzsche PC . General health checks in adults for reducing morbidity and mortality from disease. Cochrane Database Syst Rev. 2019;1(1):CD009009. 10.1002/14651858.CD009009.pub3 30699470 PMC6353639

[11] Pathak R , Kang D , Lu Y , Mansuri F , Kasen S , Deng Y , et al. Should we abandon annual physical examination? A meta‐analysis of annual physical examination and all‐cause mortality in adults based on observational studies. Prev Med. 2022;161:107130. 10.1016/j.ypmed.2022.107130 35787845

[12] Liss DT , Uchida T , Wilkes CL , Radakrishnan A , Linder JA . General health checks in adult primary care: a review. JAMA. 2021;325(22):2294–306. 10.1001/jama.2021.6524 34100866

[13] Torimoto‐Sasai Y , Igarashi A , Wada T , Ogata Y , Yamamoto‐Mitani N . Female family caregivers face a higher risk of hypertension and lowered estimated glomerular filtration rates: a cross‐sectional, comparative study. BMC Public Health. 2015;15:177. 10.1186/s12889-015-1519-6 25927998 PMC4340290

[14] Kim B , Lee Y , Noh JW , Kim TH . Factors associated with health check‐up and cancer screening participation among family caregivers of patients with dementia: a cross‐sectional study. BMC Public Health. 2021;21(1):1753. 10.1186/s12889-021-11768-8 34565358 PMC8474929

[15] Nakayama G , Masumoto S , Haruta J , Maeno T . The influence of family caregivers' experience of interprofessional care on their participation in health checkups as preventive health behavior in Japan—a cross‐sectional analysis. Int J Environ Res Public Health. 2021;18(1):223. 10.3390/ijerph18010223 PMC7796015 33396716

[16] Guilabert M , Amil P , Gonzalez‐Mestre A , Gil‐Sánchez E , Vila A , Contel JC , et al. The measure of the family caregivers' experience. Int J Environ Res Public Health. 2018;15(9):2040. 10.3390/ijerph15092040 30231535 PMC6165505

[17] Wolff JL , Freedman VA , Mulcahy JF , Kasper JD . Family caregivers' experiences with health care workers in the care of older adults with activity limitations. JAMA Netw Open. 2020;3(1):e1919866. 10.1001/jamanetworkopen.2019.19866 31977063 PMC6991279

[18] Ministry of Health Labour and Welfare . Long‐term care insurance system of Japan. 2016 [cited 2020 November 3]. Available from: https://www.mhlw.go.jp/english/policy/care‐welfare/care‐welfare‐elderly/dl/ltcisj_e.pdf

[19] World Health Organization . Innovative care for chronic conditions: building blocks for actions: global report. 2002 [cited 2020 August 2]. Available from: https://apps.who.int/iris/handle/10665/42500

[20] OECD . Health check‐ups in Japan. 2019 [cited 2020 August 2]. Available from: 10.1787/9789264311602-7-en

[21] Ministry of Health Labour and Welfare . Comprehensive survey of living conditions: long‐term care questionnaire survey. 2016 [cited 2021 February 15]. Available from: https://www.mhlw.go.jp/toukei/chousahyo/koku28ka.pdf

[22] Ministry of Health Labour and Welfare . Comprehensive survey of living conditions: health questionnaire survey. 2016 [cited 2020 August 2]. Available from: https://www.mhlw.go.jp/toukei/chousahyo/koku28ke.pdf

[23] Nakayama G , Masumoto S , Haruta J , Maeno T . Measuring family caregivers' experience of interprofessional care for patients and families: development of the Japanese version of the Caregivers' experience instrument. Fam Pract. 2020;37(6):854–61. 10.1093/fampra/cmaa059 32589192

[24] Mira JJ , Nuno‐Solinis R , Guilabert‐Mora M , Solas‐Gaspar O , Fernández‐Cano P , González‐Mestre MA , et al. Development and validation of an instrument for assessing patient experience of chronic illness care. Int J Integr Care. 2016;16(3):13. 10.5334/ijic.2443 PMC5350641 28435422

[25] Wagner EH , Austin BT , Davis C , Hindmarsh M , Schaefer J , Bonomi A . Improving chronic illness care: translating evidence into action. Health Aff (Millwood). 2001;20(6):64–78. 10.1377/hlthaff.20.6.64 11816692

[26] Tang YY , Chen SP . Health promotion behaviors in Chinese family caregivers of patients with stroke. Health Promot Int. 2002;17(4):329–39. 10.1093/heapro/17.4.329 12406921

[27] Reeves KW , Bacon K , Fredman L . Caregiving associated with selected cancer risk behaviors and screening utilization among women: cross‐sectional results of the 2009 BRFSS. BMC Public Health. 2012;12:685. 10.1186/1471-2458-12-685 22908937 PMC3499215

[28] Kumamoto K , Arai Y , Zarit SH . Use of home care services effectively reduces feelings of burden among family caregivers of disabled elderly in Japan: preliminary results. Int J Geriatr Psychiatry. 2006;21(2):163–70. 10.1002/gps.1445 16416464

[29] Aoki T , Fukuhara S , Yamamoto Y . Development and validation of a concise scale for assessing patient experience of primary care for adults in Japan. Fam Pract. 2020;37(1):137–42. 10.1093/fampra/cmz038 31325300

[30] Messina CR , Lane DS , Glanz K , West DS , Taylor V , Frishman W , et al. Relationship of social support and social burden to repeated breast cancer screening in the women's health initiative. Health Psychol. 2004;23(6):582–94. 10.1037/0278-6133.23.6.582 15546226

[31] Strine TW , Chapman DP , Balluz L , Mokdad AH . Health‐related quality of life and health behaviors by social and emotional support. Their relevance to psychiatry and medicine. Soc Psychiatry Psychiatr Epidemiol. 2008;43(2):151–9. 10.1007/s00127-007-0277-x 17962895

[32] Noguchi Y . Social support for the elderly: concepts and measurements. Soc Gerontol. 1991;34:37–48.

[33] Prochaska JO , Velicer WF . The transtheoretical model of health behavior change. Am J Health Promot. 1997;12(1):38–48. 10.4278/0890-1171-12.1.38 10170434

[34] Matthews JT , Dunbar‐Jacob J , Sereika S , Schulz R , McDowell BJ . Preventive health practices: comparison of family caregivers 50 and older. J Gerontol Nurs. 2004;30(2):46–54. 10.3928/0098-9134-20040201-09 15022826

[35] Prochaska JO , Redding CA , Evers KE . The transtheoretical model and stages of change. Health behavior: theory, research, and practice. 5th ed. Hoboken: Jossey‐Bass/Wiley; 2015. p. 125–48.

[36] Wang XR , Liu SX , Robinson KM , Shawler C , Zhou L . The impact of dementia caregiving on self‐care management of caregivers and facilitators: a qualitative study. Psychogeriatrics. 2019;19(1):23–31. 10.1111/psyg.12354 30088311

[37] Holt‐Lunstad J , Uchino BN . Social support and health. Health behavior: theory, research, and practice. 5th ed. Hoboken: Jossey‐Bass/Wiley; 2015. p. 183–204.

