# Supplementary material for: Family caregivers' experience with healthcare and social care professionals and their participation in health checkups: A cross sectional study in Japan
Source: J Gen Fam Med. 2022 Dec 14;24(2):110–8. doi: 10.1002/jgf2.599 (PMC10000252; doi:10.1002/jgf2.599)
Supplement: Supplementary file 1 — Appendix S1‐S2 [file JGF2-24-110-s001.doc]

**Appendix S1**

**Characteristics of municipalities**

| Municipality | Population density  (people per km^2^) | Elderly population (% of total population) |
| --- | --- | --- |
| Kasama City | 306 | 32.2 |
| Ryugasaki City | 970 | 29.2 |
| Ushiku City | 1,437 | 29.4 |
|  |  |  |
| cf. Average across all municipalities in Japan | 338 | 28.7 |

Participants were categorized into two groups based on their municipality of residence: one of the three municipalities where recruitment was conducted or other, because the National Health Insurance scheme in Japan subsidizes the cost of health checkups that are undertaken in an individual’s municipality of residence. Further, the subsidies of the three municipalities are approximately uniform. We assumed that ease of access to health checkups differed depending on whether the study participants lived in the same area as their patients (the municipalities where recruitment was conducted).

**Appendix S2**

**Sample size setting at the time the use of logistic regression analysis was planned.**

In the analysis, which comprised 26 independent variables including multiple dichotomous variables (initially not including relationship with care recipient), we estimated that a sufficient sample size would be 650,^1^ assuming that the rate of participation in health checkups was 60%.^2 3^

References

1. Peduzzi P, Concato J, Kemper E, et al. A simulation study of the number of events per variable in logistic regression analysis. J Clin Epidemiol 1996;49(12):1373-79.
2. Torimoto-Sasai Y, Igarashi A, Wada T, et al. Female family caregivers face a higher risk of hypertension and lowered estimated glomerular filtration rates: a cross-sectional, comparative study. BMC Public Health 2015;15:177.
3. Nakayama G, Masumoto S, Haruta J, et al. The Influence of Family Caregivers’ Experience of Interprofessional Care on Their Participation in Health Checkups as Preventive Health Behavior in Japan—A Cross-Sectional Analysis. Int J Environ Res Public Health 2021;18(1):223.

**The results of logistic regression analysis**

**Table** Associations of the Japanese version of the Caregivers’ Experience Instrument (J-IEXPAC CAREGIVERS) scores with family caregiver participation in health checkups (N = 629)

|  | **Bivariate model**† | ***P* value** |  | **Multivariate model**†, ‡ | ***P* value** |
| --- | --- | --- | --- | --- | --- |
|  | **Crude OR (95% CI)**§ |  |  | **Adjusted OR (95% CI)**§ |  |
| J-IEXPAC CAREGIVERS |  |  |  |  |  |
| Total score | 1.20 (1.02–1.42) | 0.025 |  | 1.23 (1.00–1.50) | 0.045 |
| Domain scores |  |  |  |  |  |
| Attention for the patient | 1.18 (1.00–1.38) | 0.049 |  | 1.17 (0.96–1.42) | 0.128 |
| Attention for the caregiver | 1.23 (1.05–1.45) | 0.012 |  | 1.26 (1.03–1.53) | 0.023 |

OR, odds ratio; CI, confidence interval

† Each score was included separately in the model.

‡ All three models were adjusted for age, gender, relationship with care recipient, self-rated health, insurance type, education, annual household income, municipality of residence, social support by relatives or acquaintances, caregiving time per day, caregivers’ experience as patients (caregivers’ PX), and participation behavior in health checkups before initiation of long-term care insurance use.

§ Per 1 SD (standard deviation) increase
